# Supplementary material for: Foraging behaviour and habitat-use drives niche segregation in sibling seabird species
Source: R Soc Open Sci. 2020 Sep 9;7(9):200649. doi: 10.1098/rsos.200649 (PMC7540780; doi:10.1098/rsos.200649)
Supplement: Supplement [file rsos200649supp1.pdf]

Supplement to

Foraging behaviour and habitat-use drives niche segregation in sibling seabird species

Ryan R. Reisinger, Tegan Carpenter-Kling, Maëlle Connan, Yves Cherel, Pierre A. Pistorius*

*Author for correspondence: ryan.r.reisinger@gmail.com

Supplementary Tables

Supplementary Table S1

Details of 101 giant petrels studied at Marion Island.

We used GPS tracks from 94 individuals. We used $\delta^{13}C$ and $\delta^{15}N$ values from the blood plasma of 90 individuals. GPS tracking and stable isotope ratio values were coincident in 75 individuals. Sex: F – female; M – male. Where culmen length was not measured, birds were sexed molecularly.

ID	Sex	Culmen length (mm)	Tracking						Stable isotope analyses					
									$\delta^{13}C$ (‰)		$\delta^{15}N$ (‰)		C:N ratio	
			Deployment date	Deployment duration (days)	Maximum distance (km)	Mean latitude	Long trips?	Short trips?	Plasma	Delipidated plasma	Plasma	Delipidated plasma	Plasma	Delipidated plasma
Northern giant petrel														
NGP01_092017	F	88.7	2017-09-30	5.0	660.6	-43.378	Y	N	-21.9	-19.4	15.6	15.4	4.74	3.63
NGP01_26092016	F	89.6	2016-09-28	9.1	1805.0	-40.528	Y	N	-21.9	-	12.7	-	4.80	-
NGP02_26092016	F	95.7	2016-09-28	11.5	1924.3	-40.572	Y	N	-21.2	-19.6	14.6	14.8	4.88	3.44

NGP02_KD_SEP_2015	F	-	-	-	-	-	-	-	-20.3	-18.4	14.8	15.3	4.71	3.45
NGP04_26102016	F	88.7	2016-10-01	7.5	1724.0	-42.619	Y	N	-23.7	-21.4	13.8	14.0	5.08	3.46
NGP06_092017	F	87.1	2017-10-04	5.9	631.2	-43.627	Y	N	-23.6	-21.3	13.9	14.0	4.75	3.54
NGP06_26102016	F	88.5	2016-09-28	10.9	539.0	-45.558	Y	N	-24.6	-21.7	13.2	13.3	5.40	3.56
NGP06_KD_SEP_2015	F	92.5	2015-09-20	9.9	1817.2	-41.612	Y	N	-20.9	-19.8	14.5	14.7	4.64	3.72
NGP07_092017	F	-	-	-	-	-	-	-	-23.7	-20.6	14.0	14.1	5.22	3.50
NGP08_092017	F	87.4	2017-10-01	6.6	1665.6	-39.379	Y	N	-22.1	-19.7	14.1	14.2	4.91	3.52
NGP08_KD_SEP_2015	F	90.4	2015-09-20	10.3	1750.7	-41.535	Y	Y	-21.4	-19.1	14.5	15.1	5.03	3.50
NGP09_092017	F	91.7	2017-09-30	4.5	1056.6	-45.002	Y	N	-22.9	-20.8	13.7	13.9	4.87	3.53
NGP09_26102016	F	90.7	2016-09-26	9.0	1754.3	-40.286	Y	N	-23.7	-20.8	14.6	14.7	5.37	3.39
NGP10_092017	F	88.9	2017-09-28	7.3	514.5	-46.469	Y	Y	-22.0	-19.9	14.5	14.6	4.85	3.80
NGP10_26102016	F	-	-	-	-	-	-	-	-20.6	-19.0	14.8	14.8	4.56	3.47
NGP10_KD_SEP_2015	F	-	-	-	-	-	-	-	-21.2	-19.2	14.6	15.1	4.62	3.52
NGP11_092017	F	92.0	2017-09-28	6.1	1413.2	-39.224	Y	N	-21.6	-19.9	14.6	14.6	4.77	3.87
NGP12_092017	F	85.3	2017-10-06	13.1	1768.6	-40.748	Y	N	-21.6	-19.4	15.0	15.0	4.78	3.77
NGP12_26102016	F	90.6	2016-10-03	20.7	234.9	-47.002	Y	Y	-23.4	-	11.9	-	4.56	-
NGP13_092017	F	-	-	-	-	-	-	-	-20.8	-18.9	14.5	14.8	4.71	3.61
NGP13_26102016	F	93.7	2016-09-28	9.2	1310.8	-43.143	Y	N	-22.0	-19.9	14.3	14.4	4.72	3.35
NGP13_KD_SEP_2015	F	92.2	2015-10-04	11.0	727.2	-46.459	Y	Y	-21.9	-20.0	13.6	14.0	4.61	3.56
NGP14_092017	F	-	-	-	-	-	-	-	-21.2	-19.5	14.1	14.0	4.44	3.54
NGP14_26102016	F	92.3	2016-10-02	7.0	1806.1	-39.720	Y	N	-21.6	-20.1	15.1	12.6	4.82	3.40
NGP15_092017	F	87.4	2017-10-06	7.3	1737.7	-39.131	Y	N	-20.0	-18.0	15.0	15.1	4.70	3.50
NGP15_26102016	F	87.2	2016-09-29	8.0	1285.5	-40.679	Y	Y	-20.5	-19.4	15.9	15.1	4.62	3.34
NGP16_092017	F	87.8	2017-09-29	5.2	519.5	-45.452	Y	N	-22.2	-19.9	14.3	14.5	4.77	3.69
NGP16_26102016	F	93.8	2016-09-27	10.9	1846.6	-40.813	Y	N	-21.0	-	14.7	-	5.31	-
NGP16_KD_SEP_2015	F	92.2	2015-10-03	9.0	1740.0	-39.754	Y	N	-20.0	-18.6	14.6	14.8	4.58	3.51
NGP17_092017	F	90.2	2017-09-27	6.8	1523.1	-38.121	Y	N	-21.6	-18.7	15.2	15.3	5.20	3.72
NGP17_26102016	F	93.5	2016-09-29	7.2	1422.3	-42.382	Y	N	-23.0	-20.9	14.0	14.1	5.21	3.49
NGP17_KD_SEP_2015	F	89.5	2015-10-04	10.4	1802.9	-44.089	Y	Y	-	-	-	-	-	-

NGP18_KD_SEP_2015	F	92.7	2015-10-06	5.4	96.7	-47.161	Y	N	-23.6	-21.5	13.2	13.7	4.65	3.50
NGP19_27102016	F	91.3	2016-10-05	7.3	1742.4	-38.703	Y	N	-20.7	-18.3	14.5	14.5	4.99	3.40
NGP20_092017	F	85.6	2017-10-06	14.7	1763.1	-42.813	Y	N	-24.9	-22.7	12.8	12.9	4.77	3.60
NGP20_26102016	F	91.3	2016-10-02	9.3	1737.3	-41.336	Y	N	-20.6	-18.7	15.0	15.0	4.81	3.39
NGP21_102017	F	-	-	-	-	-	-	-	-23.4	-21.3	13.0	13.1	4.79	3.79
NGP21_27102016	F	89.6	2016-10-05	10.8	88.0	-47.007	Y	N	-23.1	-	12.0	-	4.20	-
NGP01_KD_SEP_2015	M	100.1	2015-09-17	4.6	13.2	-46.961	N	Y	-23.6	-20.8	11.8	11.8	4.84	3.46
NGP02_092017	M	106.1	2017-09-30	4.9	42.7	-46.958	N	Y	-23.3	-21.0	12.9	12.9	4.63	3.51
NGP03_092017	M	-	-	-	-	-	-	-	-24.0	-21.6	12.3	12.2	4.5	3.61
NGP03_26092016	M	102.4	2016-09-30	7.2	8.7	-46.966	N	Y	-24.2	-21.7	11.9	12.0	4.79	3.39
NGP03_KD_SEP_2015	M	102.9	2015-09-17	10.8	1221.3	-39.413	Y	N	-21.3	-18.9	15.6	16.2	5.28	3.49
NGP04_092017	M	-	-	-	-	-	-	-	-21.4	-19.6	13.3	13.3	4.27	3.71
NGP04_KD_SEP_2015	M	99.3	2015-09-18	2.2	4.1	-46.965	N	Y	-	-	-	-	-	-
NGP05_092017	M	99.9	2017-09-30	6.9	1137.6	-46.224	Y	Y	-22.9	-20.4	13.6	13.7	4.78	3.73
NGP05_26102016	M	108.1	2016-09-28	7.9	231.3	-45.915	Y	N	-23.3	-21.2	14.0	13.9	4.60	3.40
NGP05_KD_SEP_2015	M	103.9	2015-09-22	8.0	1609.7	-42.874	Y	N	-21.9	-19.6	13.9	13.9	4.98	3.53
NGP07_26102016	M	107.6	2016-09-29	7.0	8.5	-46.962	N	Y	-23.7	-21.4	12.4	12.4	4.60	3.45
NGP08_26102016	M	109.7	2016-09-27	15.9	615.8	-47.899	Y	Y	-	-	-	-	-	-
NGP09_KD_SEP_2015	M	102.5	2015-09-24	8.4	69.2	-46.961	Y	Y	-	-	-	-	-	-
NGP11_26102016	M	113.9	2016-09-30	11.8	9.3	-46.955	N	Y	-22.4	-18.5	12.6	15.8	5.04	3.44
NGP11_KD_SEP_2015	M	107.7	2015-10-07	7.0	984.8	-43.375	Y	N	-22.0	-20.1	14.1	14.0	4.74	3.47
NGP14_KD_SEP_2015	M	110.0	2015-10-04	6.2	1424.2	-49.894	Y	N	-24.3	-21.7	11.8	12.3	4.62	3.32
NGP15_KD_SEP_2015	M	105.4	2015-10-03	7.6	25.6	-46.930	N	Y	-22.6	-20.4	13.0	13.2	5.00	3.61
NGP19_092017	M	-	-	-	-	-	-	-	-24.0	-21.2	12.9	13.0	4.76	3.50
NGP19_KD_SEP_2015	M	-	2015-10-03	5.9	42.8	-46.965	N	Y	-23.5	-21.4	11.9	12.2	4.68	3.52
NGP20_KD_SEP_2015	M	104.2	2015-10-04	8.8	26.6	-46.935	N	Y	-	-	-	-	-	-
NGP22_102017	M	107.0	2017-10-19	5.9	49.9	-46.951	N	Y	-23.4	-21.6	11.7	11.6	4.45	3.50
NGP23_102017	M	-	-	-	-	-	-	-	-23.3	-21.4	11.6	11.5	4.40	3.52
Southern giant petrel														

SGP01_03102016	F	92.4	2016-10-03	24.3	1341.3	-50.189	Y	N	-24.3	-22.5	12.7	12.8	4.72	3.52
SGP01_102017	F	89.2	2017-10-05	2.4	643.3	-49.427	Y	N	-25.2	-23.2	12.6	12.6	4.72	3.60
SGP02_03102016	F	-	-	-	-	-	-	-	-25.5	-23.3	12.6	12.7	4.59	3.42
SGP03_03102016	F	-	-	-	-	-	-	-	-25.2	-23.1	12.4	12.4	4.57	3.41
SGP04_102017	F	87.9	2017-10-04	6.7	1245.8	-49.900	Y	N	-24.9	-22.6	13.4	13.4	4.99	3.53
SGP04_KD_SEP_2015	F	92.3	2015-09-28	7.6	1389.5	-52.161	Y	N	-	-	-	-	-	-
SGP05_102017	F	84.9	2017-10-03	12.1	1524.6	-53.521	Y	N	-25.1	-23.1	12.6	12.6	4.96	3.57
SGP05_KD_SEP_2015	F	88.0	2015-10-01	16.6	1909.5	-52.419	Y	Y	-25.4	-23.0	12.7	12.7	4.68	3.52
SGP06_03102016	F	93.4	2016-10-08	10.8	1215.5	-51.097	Y	N	-24.2	-22.2	12.4	12.6	4.56	3.44
SGP07_03102016	F	93.3	2016-10-05	7.5	1676.0	-46.723	Y	N	-24.1	-22.2	13.2	13.2	4.71	3.42
SGP07_102017	F	88.7	2017-10-09	9.7	1245.3	-47.503	Y	N	-24.7	-	13.4	-	5.17	-
SGP07_KD_SEP_2015	F	85.3	2015-09-25	6.9	904.0	-49.377	Y	Y	-	-	-	-	-	-
SGP08_03102016	F	90.1	2016-10-04	10.6	846.8	-50.451	Y	N	-24.9	-22.3	12.8	12.8	4.72	3.40
SGP08_102017	F	89.5	2017-10-09	13.0	1305.6	-50.330	Y	N	-24.8	-22.9	13.0	13.1	4.97	3.63
SGP10_03102016	F	84.0	2016-10-09	7.4	992.7	-46.377	Y	N	-24.1	-21.8	13.3	13.4	4.72	3.46
SGP11_03102016	F	91.8	2016-10-12	16.4	1756.7	-53.214	Y	N	-25.6	-23.6	12.3	12.6	4.80	3.57
SGP11_102017	F	88.9	2017-10-04	22.0	2031.4	-49.128	Y	Y	-24.1	-22.1	11.6	11.8	4.78	3.69
SGP11_KD_SEP_2015	F	91.5	2015-10-07	10.4	1021.0	-49.530	Y	N	-23.8	-	12.9	-	4.64	-
SGP12_03102016	F	92.0	2016-10-14	14.9	1817.5	-55.898	Y	N	-	-	-	-	-	-
SGP13_03102016	F	91.6	2016-10-03	20.8	1572.8	-50.516	Y	N	-	-	-	-	-	-
SGP14_03102016	F	90.6	2016-10-11	17.5	2343.5	-51.459	Y	N	-24.0	-22.1	12.9	12.9	4.49	3.37
SGP14_KD_SEP_2015	F	89.9	2015-10-05	9.2	1089.0	-48.474	Y	N	-24.0	-22.4	12.5	12.5	4.28	3.47
SGP15_102017	F	90.2	2017-10-05	10.1	1095.3	-49.481	Y	N	-24.9	-23.3	12.8	13.0	4.86	3.72
SGP16_102017	F	83.0	2017-10-17	16.0	1090.7	-48.732	Y	Y	-25.0	-23.0	12.6	12.7	5.18	3.74
SGP16_KD_SEP_2015	F	89.8	2015-10-08	11.9	1669.3	-53.441	Y	N	-24.7	-22.9	12.3	12.5	4.52	3.45
SGP17_102017	F	88.2	2017-10-05	12.9	1716.9	-49.722	Y	N	-25.1	-23.1	13.1	13.1	4.71	3.53
SGP18_102017	F	89.0	2017-10-13	8.0	847.8	-48.910	Y	N	-24.0	-22.5	12.3	12.4	4.50	3.74
SGP18_KD_SEP_2015	F	87.9	2015-10-06	10.4	1573.7	-50.133	Y	N	-25.1	-	12.5	-	5.08	-
SGP20_102017	F	87.1	2017-10-06	3.6	379.7	-48.843	Y	N	-24.7	-22.9	12.6	12.7	4.64	3.67

SGP22_102017	F	84.9	2017-10-19	4.9	583.8	-47.916	Y	N	-24.9	-23.0	13.0	13.0	5.00	3.70
SGP01_KD_SEP_2015	M	-	2015-09-30	4.0	12.6	-46.964	N	Y	-	-	-	-	-	-
SGP02_102017	M	102.9	2017-10-07	11.8	1480.4	-54.205	Y	N	-25.6	-23.6	12.1	12.3	4.85	3.72
SGP02_KD_SEP_2015	M	110.3	2015-09-26	11.5	21.2	-46.964	N	Y	-23.2	-21.7	11.9	12.1	4.63	3.65
SGP03_KD_SEP_2015	M	103.4	2015-09-30	3.1	10.2	-46.962	N	Y	-23.1	-22.3	12.1	12.1	4.23	3.76
SGP06_102017	M	103.7	2017-10-06	4.9	9.6	-46.966	N	Y	-24.6	-22.6	11.9	12.0	4.92	3.61
SGP06_KD_SEP_2015	M	105.0	2015-10-01	3.6	13.2	-46.965	N	Y	-23.5	-22.1	11.8	12.0	4.63	3.59
SGP08_KD_SEP_2015	M	105.3	2015-09-28	10.1	9.7	-46.965	N	Y	-23.0	-21.3	11.2	11.2	4.43	3.40
SGP09_102017	M	-	2017-10-20	1.4	9.5	-46.952	N	Y	-25.1	-22.6	11.9	11.9	5.09	3.61
SGP09_KD_SEP_2015	M	106.4	2015-10-05	3.3	9.7	-46.965	N	Y	-23.8	-21.8	11.8	11.8	4.78	3.56
SGP10_KD_SEP_2015	M	103.7	2015-09-28	12.1	14.9	-46.962	N	Y	-23.4	-21.8	12.1	12.2	4.50	3.70
SGP12_102017	M	98.2	2017-10-08	4.3	49.7	-46.964	N	Y	-25.1	-22.3	12.6	12.4	4.98	2.70
SGP12_KD_SEP_2015	M	106.6	2015-10-06	4.1	9.5	-46.965	N	Y	-	-	-	-	-	-
SGP13_102017	M	-	2017-10-11	18.1	1468.9	-51.865	Y	N	-25.2	-22.9	12.7	12.8	4.77	3.49
SGP15_KD_SEP_2015	M	-	-	-	-	-	-	-	-24.0	-22.0	11.8	11.8	4.58	3.40
SGP17_03102016	M	117.9	2016-10-07	4.5	9.5	-46.964	N	Y	-23.1	-21.3	12.0	12.0	4.33	3.43
SGP17_KD_SEP_2015	M	101.3	2015-10-06	13.9	1735.9	-53.658	Y	N	-	-	-	-	-	-
SGP18_03102016	M	107.2	2016-10-12	4.6	9.6	-46.968	N	Y	-23.7	-21.9	11.6	11.6	4.37	3.43
SGP19_03102016	M	-	-	-	-	-	-	-	-24.2	-21.9	12.2	12.2	4.62	3.44
SGP19_KD_SEP_2015	M	105.5	2015-10-06	3.3	9.8	-46.965	N	Y	-23.6	-21.9	11.9	11.9	4.56	3.58

Supplementary Table S2

Details of the 8 environmental covariates used to characterize the habitat of giant petrels GPS-tracked from Marion Island. Distance (DIST) was not considered in the random forest models.

**Abbreviation**	**Description**	**Unit**	**Spatial resolution**	**Temporal resolution**	**Source/Reference**
CHL	Chlorophyll-a concentration<br>Chlorophyll-a concentration from NOAA MODIS Aqua (OCI Algorithm)	mg/m^3	9 km	Monthly	NASA Goddard Space Flight Center, Ocean Ecology Laboratory, Ocean Biology Processing Group. Sea-viewing Wide Field-of-view Sensor (SeaWiFS) Chlorophyll Data; 2014 Reprocessing. NASA OB.DAAC, Greenbelt, MD, USA. doi: 10.5067/ORBVIEW-2/SEAWIFS/L3M/CHL/2014.
DEPTH	Ocean depth<br>GEBCO_2019 grid	m	15 arc seconds	NA	General Bathymetric Chart of the Oceans (GEBCO) https://www.gebco.net/data_and_products/gridded_bathymetry_data/gebco_2019/gebco_2019_info.html
DIST	Distance from deployment location	km	NA	NA	NA
EKE	Eddy kinetic energy<br>Calculated as:<br>$EKE = 0.5(CURRU^2 + CURRV^2)$<br>Where:<br>CURRU is the horizontal (zonal) geostrophic velocity<br>CURRV is the vertical (meridional) geostrophic velocity<br>Both calculated from Delayed-time, multi-altimeter satellite, gridded vertical geostrophic velocity (DT-MADT-UV)	cm^2/s^2		Daily	Altimeter products were produced by Ssalto/Duacs and distributed by Aviso, with support from CNES (http://www.aviso.altimetry.fr/duacs/)
SSHA	Sea surface height anomaly<br>Delayed-time, multi altimeter satellite, gridded sea surface heights computed with respect to a twenty-year mean (DT-MSLA-H)	m	0.12°	Daily	Ssalto/Duacs altimeter products produced and distributed by the Copernicus Marine and Environment Monitoring Service (CMEMS) (http://www.marine.copernicus.eu)

SST	Sea surface temperature<br>GHRSST Level 4 MUR Global Foundation Sea Surface Temperature Analysis (v4.1)	°C	0.01°	Daily	JPL MUR MEaSUREs Project. 2015. GHRSST Level 4 MUR Global Foundation Sea Surface Temperature Analysis (v4.1). Ver. 4.1. PO.DAAC, CA, USA. https://doi.org/10.5067/GHGMR-4FJ04.
WINDU	Horizontal (zonal) wind<br>NCEP/DOE AMIP-II Reanalysis (Reanalysis-2)	m/s	1.9°	Daily	NOAA/OAR/ESRL PSD, Boulder, Colorado, USA, (http://www.esrl.noaa.gov/psd/)
WINDV	Vertical (meridional) wind<br>NCEP/DOE AMIP-II Reanalysis (Reanalysis-2)	m/s	1.9°	Daily	NOAA/OAR/ESRL PSD, Boulder, Colorado, USA, (http://www.esrl.noaa.gov/psd/)

Supplementary Figures

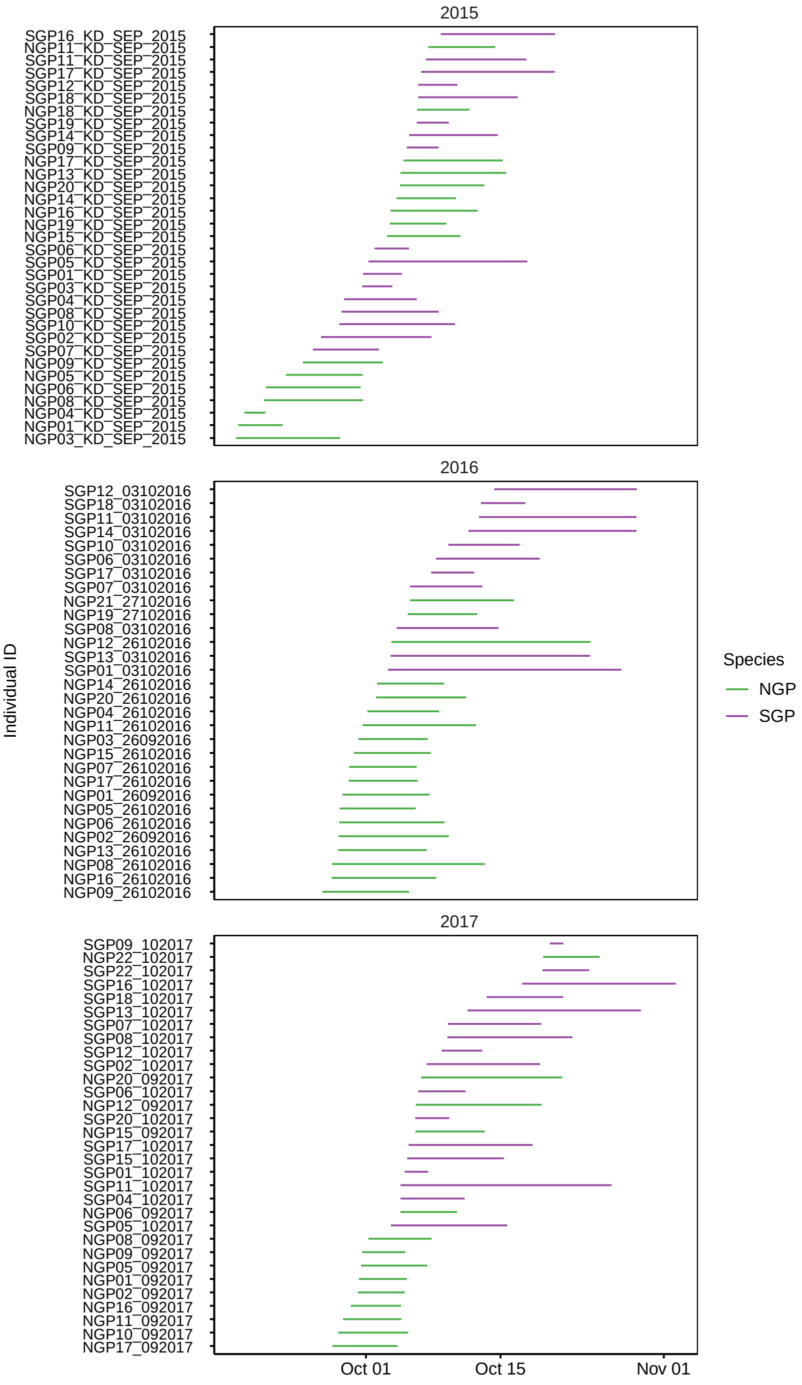

Supplementary Figure S1

Plot showing the overlapping tracking periods (horizontal lines) of 94 giant petrel individuals tracked in three years. Birds were tracked for 7.8 – 31.0 days (mean = 16.6 days). After trimming the tracks to exclude locations on the nest (shown in this figure), tracks were 1.4 – 24.3 days long, with a mean of 9.1 days

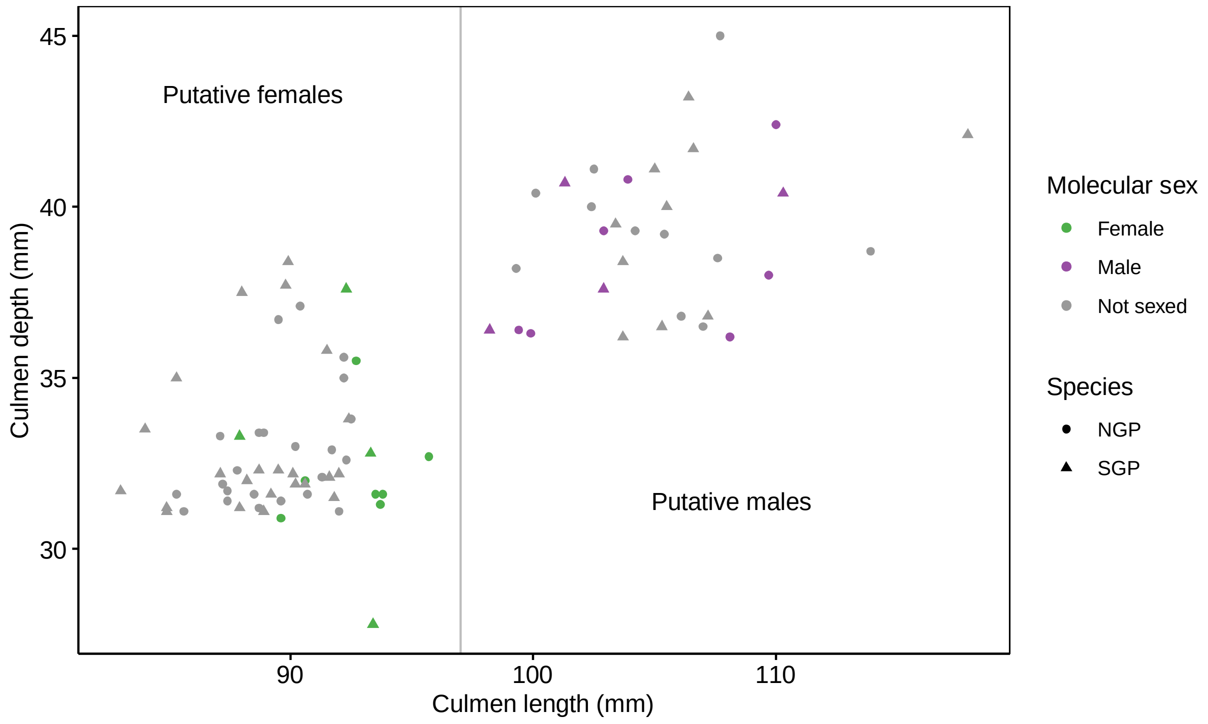

Supplementary Figure S2

Bill dimensions of 91 individuals (49 northern giant petrels [NGP] and 42 southern giant petrels [SGP]) tracked from Marion Island. The sex of 22 individuals (with bill measurements) that were sexed molecularly is indicated by different shapes. Additionally, four individuals with no bill measurements were sexed molecularly. The vertical grey line shows the 97 mm culmen length threshold that we used to distinguish putative females (< 93 mm) and putative males (> 99 mm).

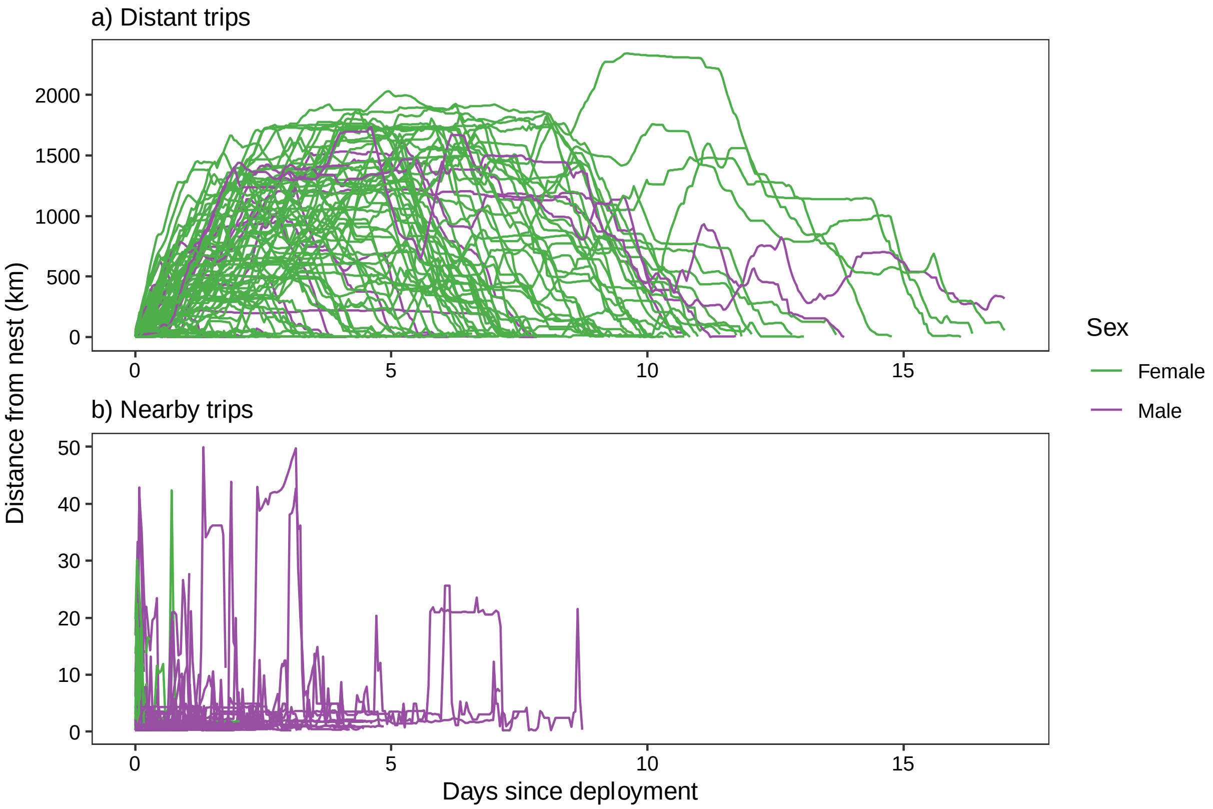

Supplementary Figure S3

Plots showing the displacement (distance from nest) against time for giant petrel individuals which made: a) distant trips (> 50 km maximum distance; 59 trips by females and 11 trips by males); and b) only nearby trips (< 50 km maximum distance; 11 trips by females and 28 trips by males). Multiple trips are defined per individual, so some individuals fall in both categories and numbers in each category do not sum to the total number of individuals (94).
